# Financial intermediation through risk sharing vs non-risk sharing contracts, role of credit risk, and sustainable production: evidence from leading countries in Islamic finance

**DOI:** 10.1007/s10668-023-03298-7

**Published:** 2023-05-08

**Authors:** Adil Saleem, Ahmad Daragmeh, R. M. Ammar Zahid, Judit Sági

**Affiliations:** 1 Doctoral School of Economics and Regional Studies, Hungarian University of Agriculture and Life Sciences, 2100 Gödöllő, Hungary; 2 School of Accounting, Yunnan Technology and Business University, Kunming, Yunnan People’s Republic of China; 3 Faculty of Finance and Accountancy, Budapest Business School, 1149 Budapest, Hungary

**Keywords:** Risk sharing, Sustainable economic output, Finance–growth nexus, Murabaha, B23, C23, F63, O12, E44

## Abstract

The asset side of Islamic banks has two different portfolios running side by side, namely risk-sharing (PLS) and non-risk sharing (non-PLS) financing. The segregation of PLS and non-PLS financing has gathered some attention recently owning to its relative importance for sustainable economic output. This study attempts to analyze the impact of decomposed Islamic financing modes (PLS and non-PLS) with a particular focus on their impact on real economic activity. In addition, we moderated the relationship with asset quality of aggregate Islamic banking sector. Quarterly data from 2014 to 2021 have been sourced from datasets of the Islamic financial service board (IFSB), the International Monetary Fund (IMF), World Bank, and Central banks’ data streams. Eleven countries have been selected based on the highest local and global share in global Islamic financial assets. Panel data regression model has been used in this study. The findings indicate that PLS financing is a weaker driver to channelize funds. However, industrial production output is significantly affected by non-PLS financing. Further the results suggest, Islamic finance–output nexus found to have a stronger relationship in the presence of higher asset quality of Islamic banks. The results show that firms mostly rely on non-PLS financing, due to reduced asymmetry and higher transparency in non-PLS contracts compared to PLS modes. The results have implications for governing bodies of Islamic financial system in boosting risk-sharing contracts and firms to limit agency conflicts arising from fluctuating cost of financing.

## Introduction

The global health crisis triggered by COVID-19 has left several economic consequences for the year 2020. Despite this pandemic, Islamic financial institutions recorded double digit growth in Islamic financial assets around the globe (IFSB, 2021). The total Islamic banking and capital assets grew by 10.7% in 2020, compared to $2.44 trillion in 2019 (IFSB, 2021). According to IFSB (2021), the growth in Islamic financial assets is witnessed across the globe, with the leading share from GCC (48.9%) followed by South-east Asia (24.9%), Middle East (20.3%) and other regions as well. It is worth noting that, compared to the growth in global assets in 2020, Islamic financial growth had been on an growth trend for the last couple of years, e.g., 9.6% in 2017–18 and 11.4% in 2019 (IFSB, 2020). The importance of growth in Islamic banking assets appeared to have catalytic role in economic growth in many countries. For instance, even at the time of financial crisis the Islamic financial industry sustained a stable growth of 18% (Ernst & Young, 2016). The observed growth in the Islamic banking industry globally in times of crisis has led investors to trust in Sharia-compliant assets. Furthermore, studies evidenced the stability (Pappas et al., 2017), effectiveness (Al-Jarrah & Molyneux, 2006), and better resilience (Mensi et al., 2020) in the Islamic financial sector in times of crisis.

The impact of the Islamic banking industry becomes manifolds if the share of Islamic banking assets in local industry is considerably higher and contributes significantly to global Islamic assets as well. Since financial development is one of the key determinants of economic growth, this phenomenon already has a strong theoretical support from supply-leading point of view (Schumpeter, 1912). In addition, there are an enormous number of studies available on analyzing the economic progress caused by conventional financial institutions (Abu-Bader & Abu-Qarn, 2008; Chaiechi, 2012; Levine, 1997; Nguyen et al., 2022; Pradhan et al., 2014; Raghutla & Chittedi, 2021). In contrast, studies argued that Islamic financial depth within the dual banking sector is healthy for promoting economic growth, through its asset-backed nature of transactions (Furqani & Mulyany, 2009; Saleem et al., 2021b). In addition, the Islamic banking–growth nexus has been the topic of discussion in many studies recently. For instance, Kassim (2016) found a significant impact of Islamic banking development and economic growth in Malaysia. Similarly, the same phenomenon has been empirically examined in Indonesia (Anwar et al., 2020), Bangladesh (Chowdhury et al., 2018), Saudi Arabia (Jouini, 2016), Pakistan (Saleem et al., 2021b), and other regions as well. Nevertheless, there are some studies arguing the contrary effect of Islamic banking on economic growth in different regions (Yüksel & Canöz, 2017; Zarrouk et al., 2017). However, in all referred studies, Islamic financial development is proxied by total financing by Islamic banks and/or Islamic banking deposits. However, there are very few exceptions where assets side of Islamic banks was decomposed into PLS and non-PLS financing (Anwar et al., 2020; Chowdhury et al., 2018). It is worth noting that in practice, the types of financing modes maintain a significant importance in the development of Shariah compliant financing products. For instance, based on the structure of business model, Islamic banks performed the dual function of financial intermediary and trading agent helping the growth of end-to-end real economic activities (Ayub, 2013; Lewis & Algaoud, 2001).

The essence of Islamic finance originated from the concept of profit and loss sharing (PLS) investments, where both the bank and customer share in profits and suffer the losses. However, there are two major dimensions of Islamic banking contracts that include participatory (PLS) and debt/sale-based contracts (non-profit and loss sharing—non-PLS) (Ayub, 2013; Lewis & Algaoud, 2001). PLS contracts are the equity-based financing, referred as the real essence of Islamic finance (Azmat et al., 2015). The objectives of Islamic Shariah (Islamic law) encourage social and economic justice by creating impartial opportunities in sharing profit and loss for both parties; these contracts shadow the true ideology of Islamic finance (Asutay, 2007; Khaled & Khandker, 2015). Furthermore, equity-based contracts are further broken down in Musharakah (partnership where both parties invest, share profits and losses), Mudarabah (partnership where one party invests in funds and other in skills), and diminishing Musharakah (diminishing partnership) (Ayub, 2013). In contrast, there are wide range of contracts that comes in debt-base financing, including Murabaha (sale with disclosed profit), Ijarah (rental agreement), Istisna (order manufacture), Salam (advance selling), Musawama (sale contract), Qard (loan without interest), and others (Khaled & Khandker, 2015). On corporate front, Islamic financial contracts proved to be a cure for agency problems, as these contracts avoid undue influence on either party and enhances the information quality (Aljifri & Khandelwal, 2013). In similar way, economically, Islamic financial contracts provide entrepreneurs the opportunity to share the risk, rather taking it all solely, in investment decisions (Kayed, 2012). As a result, there are higher chances of promoting equitable means of investment, creation of wealth, capital allocation and utilization, and economic productivity (Chowdhury et al., 2018). In addition, (Azmi et al., 2019) argued that financing from Islamic banks is more affluent for industries compared to conventional debts. Though asset side of Islamic bank is complex yet important to discuss, limited attention has been given to analyzing the relative importance of the type of Islamic financing modes and its contribution to real economic output. For instance, Bougatef et al. (2020) studied this phenomenon in the Malaysian economy by decomposing the financing types into PLS and non-PLS modes. Moreover, non-PLS financings appeared to be in a supply–growth nexus in Malaysia (Guizani & Ajmi, 2021). It is relevant to mention, Guizani and Ajmi (2021) studied investment inefficiency and role of Islamic financial contracts from corporate perspective, which is not directly linked to finance-growth nexus. On the other hand, the impact of modes of Islamic financing, i.e., PLS and non-PLS, on real economic output proxied through total industrial output is been discussed in context of Pakistan (Saleem et al., 2021a). However, the results are limited to a few geographical boundaries, and there is considerable scope for studying these modes in connection with real economic in the presence of conventional banks as well, which have been ignored in the existing literature.

This study intends to examine the effect of decomposed financing side of Islamic banking in the presence of conventional counterparts on smooth-running of economic activities. Previous studies provided evidence of the country-wide impact of decomposed financings on economic progress (Bougatef et al., 2020; Chowdhury et al., 2018; Saleem et al., 2021a). Yet ignored the presence of dual banking system, and limitation in PLS portfolios. According to the IFSB (2021) report, there are fifteen countries in the world where the Islamic banking share in the local market is more than 15%, and further contribute in global Islamic banking assets at least 1.5% (IFSB, 2021). In local markets, Iran and Sudan have 100% Islamic banking followed by Saudi Arabia with 68% (2020), Brunei at 61.9% (2020), Kuwait and Malaysia stood at 42% and 28.9% (2020), respectively. Moreover, there are countries with more than 15% contribution of Islamic banking in the local financial sector that include Qatar (27.7%), Djibouti (25%), Bangladesh (21.9%), UAE (19.0%), Jordan (17.9%), Palestine (16.5%), Pakistan (16%), and Oman (14.2%) (IFSB, 2021). It is relevant and of significant importance to study and compare the influence of PLS and non-PLS financing on real economic output in countries where Islamic banking holds significant share in the local banking industry.

The current study aims to add to the existing literature in the following ways. Firstly, knowing the fact that limited studies have been conducted in Malaysia, Bangladesh, and Indonesia. This study considers 11 major contributors of the global Islamic financial sector. The horizon of this study is wide to the countries with prominent Islamic banking industry, which would be the valuable addition to the existing body of knowledge. Secondly, to the best of authors’ knowledge, the panel of countries has been used first time in context of PLS and non-PLS financing and industrial activity. Moreover, previous studies were confined to Mudarabah and Musharakah contract only. However, element of diminishing Musharakah was completely ignored while composing PLS portfolios of Islamic banks. However, identification of participatory modes of finance can play a significant role in this context, as PLS modes are not limited to Mudarabah and Musharakah (Ayub, 2013). The extended version of Musharakah contract referred as diminishing Musharakah is also a part of the asset side of Islamic banks. Since this research considered the true conceptual structure of PLS financing (i.e., Musharakah, Mudarabah, Diminishing Musharakah) and non-PLS financing (Murabaha, Salam, Ijarah, Istisna, and others). Another important aspect that we considered the presence of dual banking system was ignored in past studies. Lastly, introduction a moderating effect of asset quality between Islamic financing modes and industrial output is one the novel contribution to the literature. A higher value of non-performing loans represents weak control and shallow compliance of Shariah products. On the other hand, higher asset quality enhances the financings to the firms which ultimately affects the overall production. Moreover, strict compliance of financing assets (ultimately firms meeting their financial obligation in time) helps the firms to maintain their credit rating as well.

## Literature review

### Islamic finance and economic growth

The theoretical basis for the causality between financial development and economic growth has been well established. There are insights available in the context of conventional financial systems, which have been empirically examined by several renowned researchers (Abu-Bader & Abu-Qarn, 2008; Chaiechi, 2012; Levine, 1997; Nguyen et al., 2022). Since countries started to implement assets backed-banking—referred to as Islamic banking—at the beginning of twenty-first century, it has led to a new debate on the Islamic finance-growth nexus. For instance, one of the seminal works on Islamic finance was documented by Furqani and Mulyany (2009), the study explored the impact of Islamic banking financing on economic growth in Malaysia using Johansen and Juselius’ cointegration and VECM model to investigate the Islamic finance–growth nexus. The authors concluded that in the long run the development in Islamic financings spurs gross domestic product and capital accumulation. However, this does not contribute to the development of trade. Similarly, Abduh and Omar (2012) argued in favor of the Islamic finance–growth nexus in the context of Indonesia by using a comparatively sound econometric model, i.e., ARDL, using quarterly banking and macroeconomic data. The authors confirmed that in the long run Islamic financings and economic growth complement one another in the long run.

Malaysia has been the subject of many empirical studies that confirmed the catalytic role of Islamic financing in spurring economic growth in the long run. Using the ARDL model and quarterly data of bank and macroeconomic variables, studies confirmed a unidirectional supply-leading relation between Islamic banking financing and economic growth (Abd. Majid & Kassim, 2015; Kassim, 2016). Moreover, Jouini (2016) showed the positive and significant impact of Islamic banking development and economic growth in Saudi Arabia by considering the annual data from 1980 to 2012. The study employed the ARDL and ECM models to test for long- and short-run dependency of economic growth on savings. Similarly, Anwar et al. (2020) posited that Islamic banking deposits and financings appeared to be prosperous for economic growth in Indonesia. Using the quarterly time series data set, the authors employed ARDL models to analyze the long-run cointegration with the development of Islamic finance and its effects on economic growth. The results indicate that Islamic banking deposits and economic growth have bidirectional causality, whereas the financings facilitate economic growth in the long run. Furthermore, the study provided insights about the intermediary role of Islamic financial industry in Indonesia, which has a positive and significant relationship with economic growth in the long run. Other studies with consistent findings highlight the importance of the Islamic financial sector and its ability to channelize funds efficiently, which ultimately corroborate economic growth in Pakistan, GCC, and the MENA region (Goaied & Sassi, 2010; Rafay & Farid, 2017; Yusof & Bahlous, 2013). Similarly, Tabash et al. (2022) argued in favor of the existence of positive causal relationship flowing from Islamic financial sector to economic growth in the context of Nigeria. The results suggested that even with weak Islamic banking infrastructure, the Nigerian economy received significant strength from Islamic banking. In addition to the time-series study, new additions to the literature on a panel of selected countries with a Muslim majority (Bahrain, Indonesia, Malaysia, and Qatar) confirmed the positive association of Islamic financial development on economic growth (Naz & Gulzar, 2022). The study was conducted on aggregated economic and financial data of selected 5 Muslim majority countries using the Panel ARDL approach. The study used three variables to represent Islamic financial development including total Islamic banking deposits, total financing made by Islamic banks, and issued sukuks. The study came up with persistent results compared to the majority of studies.

There are several recent additions to the literature involving a comparison between non-Islamic and Islamic banking, aiming to assess the relative importance of both systems. For instance, Guizani and Ajmi (2021) found significant and positive effects of the asset-backed banking industry in spurring economic growth in five GCC countries. Using Generalized and Ordinary Least square panel regression models, the authors compared the two banking systems in connection with economic growth in Saudi Arabia, Bahrain, Qatar, Kuwait, and UAE. The results indicate that the Islamic banking system is stronger and beneficial for the efficient channelizing of funds, making an economy grow. A similar finding was obtained by Saleem et al., (2021a, b) in the context of the dual banking system of Pakistan. The study employed ARDL, ECM and granger causality tests to analyze the impact of Islamic and conventional financial depth, and intermediation on economic growth. The results showed that Islamic financial depth upholds economic growth both in the long and short run. Conventional financial depth is affected in the long run but with less magnitude. Another imperative finding of this study involved the element of asset quality in measuring the financial stability of the banking system, which was found to affect economic activities negatively. Similar to previous studies, Ledhem and Mekidiche (2022)—employing an experimental approach (Markov chain marginal bootstrap resampling method) with a dual banking system—argued that the Turkish economy was no exception to experiencing a positive impact from the Islamic financial sector. The complementary effect of Islamic banking has been recently witnessed in selected countries in the MENA region (Belkhaoui, 2023). The results showed that conventional markets prevail in MENA region; however, the Islamic financial market is not only fruitful for economic growth but also for increasing demand for the conventional market. The passive role of Shariah-based banking seemed to have a positive impact on the development of the conventional financial market. Furthermore, the selection of an option by customers is primarily based on profit and reward. The religious factors amplify the existence of Islamic financial sector. Furthermore, Islamic financing has been proven to be a fuel for the economy compared to conventional financings in a period of credit crisis. Using cross-sectionally correlated and timewise autoregressive (CCTA) model, GCC countries found a positive significant relationship between the Islamic banking industry and economic growth after the credit crisis. Whereas, conventional markets were hit badly by the crunch of 2007–09 (Elmawazini et al., 2020).

In addition to the linearity in models, Mensi et al. (2020) found a nonlinear relationship between Islamic banking financings and economic growth. Authors used dynamic panel quantile regression and panel smooth transition models in 15 MENA and non-MENA countries and found that Islamic banking financing had an asymmetric relationship with economic growth. Furthermore, results showed that in most cases Islamic banking development led to economic growth across quantiles. Further evidence of nonlinearity was analyzed by Ullah et al. (2021) in Pakistan. The NARDL results suggest that an increase in Islamic banking financing leads to improved growth, whereas a decrease in financing causes a reduction in industrial production. It imperative to discuss the literature that provided contrary results to these studies. Such as Yusof and Bahlous (2013) and Zarrouk et al. (2017) who argued that asset-backed banking does not complement economic progress.

### Risk-sharing and non-risk sharing modes of financing

In sum, there are rich sources in the literature confirming the positive association of Islamic financial industry and economic growth. However, to the best of authors’ knowledge, the majority of the studies have been conducted by taking total deposits or/and total financing made by Islamic banks to represent the development of Islamic banking in an economy. However, economic growth is measured mostly through GDP, GDP per capita or GDP rate with few exceptions of the Industrial production index. Considering the essence of Islamic banking, there are few studies that corroborated the risk-sharing and non-risk sharing financing and its relation to real economic output proxied by industrial production. For instance, a seminal work in decomposed financing was that of Chowdhury et al. (2018). The study posits that risk-sharing/PLS financing positively correlates with economic growth, whereas non-PLS modes do not contribute enough to economic growth in Bangladesh. However, the study corroborated the factor of PLS and non-PLS financing in context of Bangladesh. Also, selection of PLS portfolio is made based on Musharakah and Mudarabah contracts. In contrast, Bougatef et al. (2020) found that economic activities are mainly derived by non-PLS modes in the long and short run, whereas PLS financing does not contribute neither in long not in short run. Another study confirms the relative importance of non-PLS financing in nurturing industrial production in the context of Pakistan (Saleem et al., 2021a, 2021b). In addition to the recent literature, another country-wide study (Masrizal & Trianto, 2022) suggested that PLS financing has had a larger impact in Indonesian economy. Using time series data, authors ran ARDL bounds, and results showed that PLS Islamic financing contracts are positively associated with industrial output of Indonesia. Furthermore, it was highlighted Indonesian Islamic banking industry enjoys larger share of PLS financing, i.e., above 40%. However, authors further implicated that further step should be taken by regulatory bodies to support Islamic banking industry.

On the corporate front, the investment and financial decisions are exposed to potential risks of agency issues that may have be due to changes in interest rates, payment plans, deviation from mainstream financial obligation, and, most importantly, information asymmetry. That is where Islamic banking products outweigh the interest-based debt structure with its solid governance and transparency. Strong governance and transparent featured products anticipate reductions in agency conflicts (Alam et al., 2020). Furthermore, in addition to corporate governance measures, Islamic banks maintain a dual governance structure in the form of a Shariah supervisory board (SSB) (Grassa, 2013). The emphasis is not only made on corporate governance but on the participation of SSB members in ensuring each product is confidential, independent, consistent, ethical, and transparent. It is worth noting that the added value of dual governance helps the Islamic financial sector to reduce information asymmetry and enhance transparency (Grassa et al., 2018). Therefore, the transparent contract is the key pillar of an Islamic financial institution. Every second financing product is different from the next. For instance, taking participatory contracts, where banks extend finances to corporate clients based on their credit worthiness and assurance of entrepreneurial abilities (Chowdhury et al., 2018). This gives rise to equitable means of wealth creation as well as optimal utilization of finances, rather than the adverse selection of debt-based instruments from conventional counterparts (Hassan et al., 2022). According to Kayed (2012), in PLS contracts, the risk of undertaking the investment project is replaced by risk-sharing, compared to risk-bearing. Therefore, profit sharing provides a fundamental base for starting equitable and transparent partnerships with Islamic banks (Ahmed & Aassouli, 2022). Other Islamic modes, i.e., non-PLS/sale-based, are more specific in defining terms and fixing the price of the product. This is because the contract becomes void if there is uncertainty (referred as gharar). As a result, non-PLS modes are better options for the firms as they resolve the agency issues and adverse selection of interest-based instruments (Alqahtani, 2016; Ebrahim & Sheikh, 2016; Shaban et al., 2014; Ahmed & Aassouli, 2022).

Financial intermediation and channelizing of funds have been point of discussion in the literature. Firms maintain their capital needs via several ways, and banks are one of them. Companies prefer to have symmetric information for meeting their financing needs, which help them in maintaining a healthy agency relationship as well as maximizing the shareholder’s return. Intrinsically, the financing side of Islamic banks carries both the features that eventually attracts corporate sector. For instance, PLS financing promotes equal distribution of resources, sharing of equity, profits, and losses as per efforts put by both parties to maximize their returns. On the other hands, sale-based Islamic contracts (non-PLS) address asymmetric problem of cost of financing by fixing the cost of financing through different Islamic financing contracts. On the conventional side, interest-based contracts are unable to provide shield to protect agency issues or to have an equitable entrepreneurial opportunity to maximize the productivity. Based on mentioned arguments, we built the following hypothesis:

#### H1

PLS financing has positive significant impact on industrial output

#### H2

Non-financing has positive significant impact on industrial output

### Credit risk and financial constraints

Financial sector plays a role of a heart in an economy by pumping the money supply to the entrepreneur, manufacturers, companies, and other investors. Financial intermediation is a broader concept than just circulation of money. It requires strict scrutiny and greater energy to find prime customer who can assure the repayment with profit (Power, 2007). The role of credit scrutiny is even tighter in Islamic financial products where each type is linked to a specific asset. The sustainability and stability of financial sector is not only concerned by the banking companies but also by the regulatory bodies, manufacturing sector, customers, and other stakeholders. Furthermore, better performance of assets may attract many potential clients due to higher stability (Suyanto, 2021). Therefore, the loan growth of the banking sector is un-controversially affected by financial deepening and strict compliance (World Bank, 2019). However, lending growth relies on financial liberalization and banking reforms, which at some point is negatively affected by the selection of subprime customers that gives rise to weaker asset quality (Aysan & Ozturk, 2018). Similarly, the higher non-performing assets (herein after NPA) negatively affect bank’s profitability (Epure & Lafuente, 2015; Das & Uppal, 2021; Hunjra et al., 2020; Musneh et al., 2021; Ahmed et al., 2022), which enhances the financing cost, and reduces the supply of future credits (Aiyar et al., 2015). The private sector predicts the future credit default of banking companies and uses it as a driving force to make decisions and gain confidence in expanding or contracting their credit exposure in future (Bordalo et al., 2018). Likewise, setting a sound credit policy is cyclical in nature that not only helps banks to amplify their profits but also to maintain a good reputation for gains that trigger higher lending growth (Rajan, 1994). Despite seriousness of the matter, there is no such theory that supports the higher NPAs and lower credit allocation (Accornero et al., 2017; Zahid & Simga-Mugan, 2019). In addition, it does not matter how sufficiently capitalized or profitable the banks are, credit risk significantly affects the monetary policy mechanism too (Thornton & Di Tommaso, 2021). Since it is a burden for both the lender (bank) and borrower (customer), which reposits the valuable collateral and unclaimed amount of debt. Therefore, the firms face financial constraints (Bernanke et al., 1999). A higher credit risk affects the bank’s profitability, contract the credit supply, higher financial limitations, weaken the market forces, and ultimately affects the economic growth (Balgova et al., 2016; Cucinelli, 2015). According to Mensi et al. (2020), Islamic banks have better asset quality than conventional banks due to their asset-backed nature and adherence to Shariah principles. A lower non-performing asset (NPA) is expected to intervene the relationship between Islamic financing and economic output. There are two possible reasons for that; first, a lower NPA would likely to increase the credit supply to the existing as well as the new corporate clients (Thornton & di Tommaso, 2021). Second, as better asset quality indicates companies' ability to fulfill their obligations and paying in time, which help the firms to improved credit ratings and reduces financial constraint (Bernanke et al., 1999). Based on above argument, it is expected that better asset quality will have a moderating effect on the relationship between Islamic financing and economic output (Table 1).

**Table 1:** Description of the existing literature, findings, and model used

Authors	Market scope	Independent variable	Econometric model
Furqani and Mulyany (2009)	Malaysia 1997–2005	Islamic financing (total financing) contributes to economic growth (measured through GDP per capita and GFCF)	Vector error correction model (VECM^b^) and Granger Causality test
Abduh and Omar (2012)	Indonesia 2003–2010	Islamic financing growth (total financing) spur economic growth measure by GDP and capital accumulation	ARDL^a^ and ECM^d^
Abd. Majid and Kassim (2015)	Malaysia 1997–2009	Islamic financial growth (total deposits and finance) contributed to economic growth	ARDL^a^ Bounds testing, ECM^d^, and VDC^c^
Kassim (2016)	Malaysia 1998–2013	In both long and short run Islamic banking (total deposit, and financing) positively affect economic progress (measured by industrial production)	ARDL^a^ Bounds and ECM^d^
Jouini (2016)	Saudi Arabia 1980–2012	Financial development (Credit to private sector) affects economic growth measured by (GDP)	ARDL^a^, Johansen Cointegration, ECM^d^
Goaied and Sassi (2010)	16 MENA Countries 1993–2005	Development in Islamic financial system (private credit by Islamic banks) has a weak but positive relationship with GDP	GMM^f^ panel data approach
Yusof and Bahlous (2013)	GCC and East Asia (Malaysia and Indonesia) 2000–2009	Islamic finance (total financing by Islamic banks) helps economic growth (GDP per capita) in positive direction	Panel cointegration model and VDC^c^
Rafay and Farid (2017)	Pakistan 2003–2015	Islamic finance (measured by total deposit and financing) is healthy for real economic output (proxied by Industrial production)	Johansen Cointegration, Granger Causality, Impulse response, and ECM^d^
Guizani and Ajmi (2021)	Malaysia 2007–2017	Non-risk sharing contracts play significant role in increasing investment efficiency. PLS contracts do not contribute to corporate financial decisions	Panel GMM^f^ estimation
Elmawazini et al. (2020)	GCC (5 countries) 2001–2009	Both Islamic and conventional banking systems happened to be healthy for economic growth. However, results contradict with single-country study	Panel data approach (Fixed effect, LSDV)
Chowdhury et al. (2018)	Bangladesh 1984–2014	PLS financing affects economic growth positively and non-PLS financing negatively affects the economic growth of Bangladesh	ARDL^a^, VAR^e^ model
Bougatef et al. (2020)	Malaysia 2010–2018	Non-PLS financing helps industrial production to grow; however, there is no significant relationship between PLS contracts and economic progress (industrial production)	ARDL^a^ and Toda and Yamamoto causality test
Saleem et al. (2021a)	Pakistan 2005–2019	Non-PLS financing contracts facilitate real economic activities (measures by Industrial production)	ARDL^a^, VECM^b^
Masrizal and Trianto (2022)	Indonesia 2009–2018	PLS financing has larger impact on Indonesian economic growth	ARDL^a^

^a^Auto-Regressive Distributive Lag, ^b^Vector Error Correction Model, ^c^Variance Decomposition, ^d^Error correction model, ^e^Vector Auto Regressive, ^f^Generalized Methods of moments

#### H3

Better asset quality positively moderates the Islamic finance and aggregate economic output relationship

## Methods and materials

Our initial sample included 15 countries with contribution to global financial assets up to 10%. According to annual report published by Islamic Financial Service Board (IFSB, 2021), 15 countries that have double digit growth in local as well as in international Islamic financial market. The rationale of sample selection was to analyze those country with larger share of Islamic financial sector. Locally, Iran and Sudan have full-fledge Islamic banking (100%), there is 68% in Saudi Arabia, 61.9% in Brunei Darussalam, 42% in Kuwait, 28.9% in Malaysia, 27.7% in Qatar, 25% in Djibouti, 21.9% in Bangladesh, 19% in UAE, 17.9% in Jordan, 16.5% in Palestine, 16% in Pakistan, and 14.2% in Oman (IFSB, 2021). In addition, countries with leading Islamic banking share also contribute to the global Islamic market share up to 10–15%. However, we looked for quarterly data and preferred a balance panel dataset. We dropped 4 countries (Egypt, Brunei Darussalam, Qatar, and Bahrain) due to the non-availability of data. This resulted in a balance panel from 2014 to 2021 with quarterly frequency focusing on 11 countries: Saudi Arabia, Oman, Jordan, Malaysia, Indonesia, Pakistan, Bangladesh, Kuwait, UAE, Iran, and Kazakhstan. Data related to Islamic banking are sourced from the Islamic Financial Service Board (IFSB) database and respective countries’ central bank data streams. However, macroeconomic variables were extracted from International financial statistics (IFS) and the World Bank (WDI banking) database. Furthermore, for Kuwait, Iran, and Bangladesh quarterly bank specific data were extracted from the central bank of Kuwait, the statistical center of Iran, and the Bangladesh bank (central bank of Bangladesh), respectively. The Islamic Financial Service Board (IFSB) is an international regulatory body that develops and implements the standards relating to Islamic finance. It also supports the research and financial soundness of Islamic financial institutions around the globe. IFSB extracts a comprehensive dataset from respective central banks, which includes an aggregated financing side of Islamic banks and Islamic windows as well. Furthermore, it also conforms to the Basel I, II, and III requirements. To maintain uniformity, we gathered the data in USD.

### Variable constructions

#### Dependent variable

Total industrial production is considered as a proxy for real economic activity (following (Bougatef et al., 2020; Masrizal & Trianto, 2022)). Industrial production is one component of GDP, which is directly connected to bank financings and deposit. Firms are more likely to go for financing in order to have a stable capital structure and enjoy a certain level of financial leverage. Therefore, the financial sector has a direct impact on manufacturing concerns. With the advancement of Islamic banking, the concept of risk sharing has evolved in the past two decades. Islamic financing contracts leverage the firms with better transparency and limit agency problems (Alqahtani, 2016; Ebrahim & Sheikh, 2016; Shaban et al., 2014). As per the above discussion, industrial production signifies an accurate proxy for real economic activities.

#### Independent variables

Following the findings of Bougatef et al. (2020) and Guizani and Ajmi (2021), the financing side of Islamic banks in our sample is decomposed into risk-sharing (PLS) financing and non-risk sharing (non-PLS) financing.

#### PLS financing

Based on our dataset, we collected comprehensive data on the asset side of all Islamic banks and Islamic windows in selected countries, including Murabaha (sale on disclosed profit), Tawarruq (liquidity contract, buying on credit and selling in cash), Salam (advance selling), Istisna (order to manufacture), Ijarah (rental), Musharakah (partnership), Mudarabah (partnership with services and capital), Diminishing Musharakah (declining capital partnership), Qard (interest free loan), and other modes. We formulated PLS financing by grouping Musharakah, Mudarabah, and Diminishing Musharakah. In the previous literature (Bougatef et al., 2020; Guizani & Ajmi, 2021; Masrizal & Trianto, 2022), diminishing Musharakah was completely ignored. Hence, by nature, it is one of the risk-sharing modes of financing in Islamic banks (Ayub, 2013).

#### Non-PLS financing

All debt/sales-based contracts are combined to represent non-PLS financing. The Islamic Shariah does not permit selling anything without fixing the price. Islamic banks, while making non-PLS contracts, fix the final price to remove uncertainties (Gharar) and enhance transparency in these contracts. According to Alqahtani (2016) and Shaban et al. (2014), non-PLS modes of financing reduce the cost of agency. Compared to conventional debt-based contracts, firms prefer to choose Islamic modes to mitigate agency problems.

#### Moderating variable

Asset quality is one of the key determinants of sustainability of the financial sector and their performance. We considered non-performing assets (NPAs) by measuring the non-performing loans/total financing. The lower the NPA ratio, the better the asset quality of Islamic banks. Hassan and Hussein (2003) and Mensi et al. (2020) argued that, due to asset-backed nature and strict compliance of Shariah principles, Islamic banks enjoy better asset quality compared to conventional banks. Similarly, it is expected that a lower NPA would strengthen the Islamic financing–economic output relationship. A lower NPA refers to the responsibility of companies to meet their obligation that will result in better credit rating and hence fewer financial constraints for the firms. We anticipate that better asset quality intervenes and moderates the Islamic financing and economic output relationship.

#### Control variables

Following the studies (Zarrouk et al., 2017; Rafay & Farid, 2017; Pappas et al., 2017; Nguyen et al., 2022; Mensi et al., 2020; Bougatef et al., 2020; Guizani & Ajmi, 2021, Masrizal & Trianto, 2022), we controlled our model with country-specific control variables. We used imports and exports as a proxy of trade openness. More imports are expected to have a negative impact on industrial output, as it discourages local production and people rely more on imports. On the other hand, exports reflect the ease of doing business. More exports would indicate higher industrial output, more revenue, which consequently supports growth in the industrial sector (real economic sector). Gross fixed capital formation (GFCF) is used to represent capital accumulation, which complements the corporate sector and enhances output (Suri & Chapman, 1998). Furthermore, we also controlled our analysis for inflation, as inflation is one of the key determinants of economic activity in a country. If the inflation threshold effect is not present, we expect inflation to have a negative effect on industrial output as long as it stays below 3% (Khan & Senhadji, 2001). The model is also controlled by taking conventional financial depth. Loans to the private sector as a percentage of GDP are taken as a proxy of conventional financing. Although Islamic finance experienced tremendous growth in previous decades, the prevalence of Islamic financing compared to interest-based banking still seems low. Despite this, the countries under study have an Islamic banking share from 100% to the minimum 25%, within the country itself. Therefore, we considered the existence of interest-based banking parallel to Islamic banking to produce robust results. Furthermore, for robustness analysis, we also considered total deposits of the Islamic banking sector.

Table 2 provides the list of variables, abbreviations used, sources of data collection.

**Table 2:** Variable used

Variable	Abbreviation	Data source
Industrial production	IP	International financial statistics (IFS)
Profit and loss sharing	PLS	Financial service board (IFSB)
Non-profit and loss sharing	Non-PLS	Financial service board (IFSB)
Conventional financing	CF	World bank dataset (WDI)
Exports	Ex	World bank dataset (WDI)
Inflation	Inf	World bank dataset (WDI)
Capital formation	GFCF	World bank dataset (WDI)
Non-performing assets	NPA	Financial service board (IFSB)
Imports	Im	World bank dataset (WDI)

### Econometric Specification

Based on the nature of the dataset, i.e., balanced panel and previous studies (Elmawazini et al., 2020; Guizani & Ajmi, 2021; Lebdaoui & Wild, 2016; Ledhem & Mekidiche, 2022; Yusof & Bahlous, 2013), a panel regression model is used in this study. Unlike dynamic panel models, following Hayakawa’s (2007) approach, the selected dataset could not fulfill the prerequisite of a larger number of individuals (countries in this study) and a small and finite sample (number of periods). Therefore, we employed an ordinary least square regression with country and year as a fixed effect (following (Baltagi, 2013; Hsiao, 2014)).

Estimating the growth model using endogenous growth is critical when it comes to the selection of control variables. The issue of selection of control variables is resolved by open-ended hypotheses (Brock & Durlauf, 2001) that develop the causal relationship between economic growth and the selected variable to estimate the model. Similarly, Durlauf and Quah (1999) have highlighted almost 90 control variables that are potentially associated with economic growth variables. The objective of this study is not to account for all theories of economic growth but to analyze the impact of PLS and non-PLS financing in achieving sustainable production by industries. Therefore, based on above arguments and previous studies (Lebdaoui & Wild, 2016; Ledhem & Mekidiche, 2022), the effect of risk-sharing and non-risk sharing financing on industrial output is framed as an “endogenous growth model” as represented in Eq. (1)

. We employed clustered (country level) robust standard errors and a fixed effect model. Panel regression models are considered efficient for producing reliable results with a high degree of freedom (Balls & O’Donnell, 2002; Baltagi, 2013; Hsiao, 2014). Based on the Chow test and Hausman test (Baltagi, 2013; Hausman, 1978), a fixed effect model has been chosen. Equation 1 represents the base model.

1 $${y}_{it}= {\alpha }_{0i}+ {\beta }_{1}{X}_{i.t}+{\beta }_{2}{Z}_{i.t}+{\beta }_{3}{X}_{i.t}*{NPA}_{i.t}+{\varepsilon }_{it}$$

${y}_{it}$ is the dependent variable, which is the log of total industrial output of $i$ country at time $t.$ ${X}_{i.t}$ represents the independent variable, in this case we employed two separate models. The first model examines the impact of PLS-financing (log of PLS) on real economic output. In the second model, non-PLS (log) financing is modeled with the same set of control variables (*Z*_*i.t*_), i.e., conventional financing depth (CF), inflation (Inf), imports (Im), exports (Ex), and gross fixed capital formation (GFCF). Furthermore, we added an interaction term between independent variables and asset quality to examine how NPA intervenes in the relationship. All the variables are time-variant by nature at time ($t$) and country ($i$).

## Results and discussion

### Descriptive statistics

Table 3 shows the descriptive statistics of the variables. Since we adopted a balanced panel, the total number of observations are the same for each variable. Industrial production (IP) shows a mean value of 23.73 with a slight standard deviation, which shows that industrial output in a given period has not fluctuated as lot. It also shows constant growth in industrial output over the given period. On the financing side, PLS-financing appears to have more deviation from the mean compared to non-PLS financing. It indicates that a majority of countries have greater focus on non-PLS financing than risk-sharing contracts. Also, the higher deviation in PLS contracts indicates that there are countries who have less focus on PLS modes. On the other hand, conventional financial depth is an unstable variable with a big gap between min 8.86 and max 62.58 value. We suggest that the countries with the least share of Islamic financing have a larger share of interest-based financing and vice versa. Inflation is quite stable, and, as per this data, we expect it to see healthy inflation for economic progress. Exports, Imports, and GFCF all follow a similar trend. Moreover, the mean value of Asset quality is 3.8%, with a minimum value of 0%. It implies that Islamic banks are efficiently managing their financing side with strict Shariah compliance to attain fewer NPAs.

**Table 3:** Descriptive statistics

Variable	Obs.	Mean	Std. dev.	Min.	Max.
IP	396	23.733	1.179	21.197	25.551
non-PLS	396	12.833	2.32	8.283	18.232
PLS	396	9.888	3.803	.693	15.998
CF	396	25.966	12.671	8.86	62.580
Ex	396	9.835	1.014	7.35	13.729
Inf	396	.011	.02	− 0.03	.156
GFCF	396	9.416	1.13	6.95	11.38
NPA	396	.038	.032	0	.143
Im	396	9.704	.867	8.204	11.302

### Correlation matrix and multicollinearity

A pairwise correlation matrix and multicollinearity tests are provided in Table 4. Both non-PLS and PLS have been found to have a positive and significant correlation with the dependent variable. The magnitude of correlation for both financing modes is not high compared to CF. However, the results of PLS and non-PLS are as expected. NPA is negatively correlated, which shows that better asset quality would increase the output in the end. In contrast, modes of financing are strongly correlated with each other with a correlation of 0.759. We suggest that there is a growing trend in both the modes as Islamic banking grows over time. Although a high correlation might be an indication of multicollinearity, none of the coefficients among the independent variables reached 0.80. In relation to this, Table 4 provides the VIF and tolerance levels of all variables. The general rule regarding the presence of multicollinearity is indicated by a value of VIF exceeding 10 (O’brien, 2007). The variables under study have a maximum VIF of 5.744, in case of Imports, as Imports, Exports and GFCF seem to have a high correlation. As per the VIF, our model is unencumbered with a multicollinearity issue.

**Table 4:** Pairwise correlations and Multicollinearity results

Variables	(1)	(2)	(3)	(4)	(5)	(6)	(7)	(8)	(9)		VIF	1/VIF
(1) IP	1.000									Im	5.744	0.174
(2) non-PLS	0.185***	1.000								PLS	5.560	0.180
	(0.001)											
(3) PLS	0.269***	0.759***	1.000							CF	5.124	0.195
	(0.000)	(0.000)										
(4) CF	0.595***	0.254*	− 0.115	1.000						n-PLS	4.372	0.229
	(0.000)	(0.051)	(0.091)									
(5) NPA	− 0.100	0.126**	0.452**	− 0.614***	1.000					Ex	4.322	0.231
	(0.079)	(0.027)	(0.050)	(0.001)								
(6) Ex	0.844***	0.161***	0.229***	0.068	0.021	1.000				GFCF	3.744	0.267
	(0.000)	(0.005)	(0.000)	(0.231)	(0.150)							
(7) Inf	0.038	0.359***	0.434***	0.490***	− 0.099*	− 0.475***	1.000			NPA	1.906	0.525
	(0.503)	(0.000)	(0.000)	(0.000)	(0.081)	(0.001)						
(8) GFCF	0.785***	0.428*	0.581***	0.221***	0.689**	0.118**	0.315**	1.000		INF	1.600	0.625
	(0.000)	(0.000)	(0.000)	(0.000)	(0.000)	(0.038)	(0.021)					
(9) Im	− 0.749***	0.022	0.213***	0.108*	0.855**	− 0.143**	− 0.727***	0.682**	1.000	Mean	4.065	–
	(0.000)	(0.707)	(0.000)	(0.058)	(0.000)	(0.012)	(0.000)	(0.006)		VIF		

***p < 0.01, **p < 0.05, *p < *0.1*

### Regression results

Table 5 shows the estimated regression for both PLS and non-PLS models. The results for PLS financing are provided in columns (1) to (4), and results for non-PLS and IP are shown in columns (5) to (8). The analysis is initiated with a univariate model between financing sides and its impact on industrial production. At the beginning, we did not fix the year and country effect. Univariate estimates for PLS financing are presented in column (1), which is positive and statistically significant at 1%. Likewise, estimates from non-PLS model are presented in column (5), and the results are according to our expectations. To produce robust estimates, we developed our model through steps to ensure the effect of other control variables. Hence, as a next step, we added the control variables in both the models with fixing the effects of year and country. For the PLS model with control variables (country and year fixed effect), the results are given in column (2) and column (6) for non-PLS modes. We found consistent results in the second regression model as well. Furthermore, we included the moderation effect of asset quality (NPA). We introduced an interaction term between financing and NPA, to capture the moderation effect of asset quality. The results are depicted in column (3) and (7) for PLS and non-PLS, respectively. The estimates in both the models gave positive significant results.

**Table 5:** Regression results

	(1)	(2)	(3)	(4)	(5)	(6)	(7)	(8)
Variables	IP	IP	IP	Robustness check	IP	IP	IP	Robustness check
		PLS				non-PLS		
PLS	0.0834***	0.0699***	0.00232*	0.00828*	–	–	–	–
	(4.885)	(6.440)	(1.981)	(1.974)				
Non-PLS	–	–	–	–	0.0938***	0.0905***	0.137***	0.154***
					(3.285)	(5.443)	(6.708)	(7.914)
Ex	–	0.749***	− 0.0221	0.00102	–	0.827***	0.0353	0.0514
		(13.37)	(− 0.644)	(0.0330)		(14.17)	(1.273)	(1.414)
Inf	–	6.471***	2.987***	− 0.307	–	4.547***	1.411***	− 1.238***
		(3.940)	(5.762)	(− 0.511)		(2.851)	(3.254)	(− 3.487)
CF	–	− 0.517**	0.435**	0.522***		0.711***	0.543***	− 0.479***
		(− 2.014)	(2.207)	(3.377)		(4.254)	(3.544)	(− 5.224)
Dep	–	–	–	− 0.0350	–	–	–	0.0302
				(− 1.608)				(0.113)
GFCF	–	0.594***	0.278***	0.401***	–	0.550***	0.185***	0.300***
		(12.15)	(4.039)	(6.356)		(11.50)	(3.367)	(5.370)
Im	–	− 0.207***	− 0.00144	0.00969	–	− 0.309***	− 0.00155	0.00342
		(− 2.870)	(− 0.0435)	(0.327)		(− 3.866)	(− 0.0587)	(0.150)
NPA	–	–	− 13.94***	− 11.31***	–	–	–	–
			(− 3.381)	(− 3.060)				
PLS*NPA	–	–	0.772**	0.635**	–	–	–	–
			(2.218)	(2.040)				
NPA	–	–	–	–	–	–	− 32.40***	− 24.20***
							(− 8.438)	(− 6.061)
Non-PLS*NPA	–	–	–	–	–	–	2.228***	1.682***
							(7.777)	(5.991)
Constant	22.91***	13.39***	22.62***	21.33***	22.53***	14.53***	21.04***	19.75***
	(126.7)	(40.32)	(30.11)	(31.00)	(60.48)	(37.98)	(32.66)	(27.61)
Year effect	No	Yes	Yes	Yes	No	Yes	Yes	Yes
Country effect	No	Yes	Yes	Yes	No	Yes	Yes	Yes
Observations	396	396	396	440	396	396	396	440
R-squared	0.072	0.520	0.687	0.790	0.034	0.513	0.692	0.794
F-value	23.86***	273.4***	1332***	1581***	10.79***	261.9***	2090***	6741***
LM testProb. > ${\chi }^{2}$	–	–	0.079			0.156	–	–
Breusch-Pagan	–	–	0.216	–	–	0.114	–	–
Hausman Test			18.647			23.088		
			(0.005)			(0.001)		

***p < *0.01*, **p < *0.05*, *p < *0.1*

In addition, for a robustness check, we introduce a measure of savings by using total deposits held by financial sector in selected countries. Savings and investments play an incredible role in fostering economic activities (Ribaj & Mexhuani, 2021). People tend to consume less in cases of greater saving, which could result in more invested capital, consequently triggering the economic activities on an aggregate level (Omoregie & Ikpesu, 2017). In column (4) and (8), we added deposits to represent an additional control element to ensure that our estimates are robust.

### PLS and non-PLS financing

The coefficient of PLS financing in all of our estimated models (1)–(4) is positive and statistically significant. However, the magnitude of the effect kept decreasing as we added more variables. In column (3), PLS is 0.00232 and significant at 10%. However, the effect was stronger in the univariate model (1), i.e., 0.0834 significant at 1%. This indicates that PLS financing is one of the influencing factors in promoting real economic activities. However, the impact is weak due to its relative depth in the economy. Parallel to PLS impact on IP, we found a rather stronger, positive and significant effect of private credit by the conventional banking sector, which is 0.435 significant at 5%. On the other hand, we found that non-PLS financing facilitates the real economic activities with greater strength. As the results show, the coefficient of non-PLS 0.094, 0.091, and 0.137 in model (5) to (7), respectively, got stronger in magnitude, also significant at 1%. Compared to PLS, non-PLS appeared to have a larger influence over industrial output. Moreover, the impact of CF is also as we expected, i.e., positive (0.543) significant at 1%. This result is consistent with the findings of Bougatef et al. (2020) and Saleem et al. (2021a) with plausible reasoning for a relatively smaller share of risk-sharing financing in total Islamic financings. Our findings also uncovered elements contradicting the findings of the existing literature and produced significant results at 10% for PLS financing. It is pertinent to mention that prior studies (Bougatef et al., 2020; Guizani & Ajmi, 2021) focused on one country’s (in this case, Malaysia) time series aggregated data. On average, PLS contributes up to 10–15% of total Islamic financing side in Malaysia. We produced reliable and robust results with the inclusion of a majority of countries where PLS share is over 50% (Pakistan, Iran, and Indonesia) (IFSB, 2021). Another reason for experiencing the weaker impact of PLS is that risk-sharing financing is often discouraged by Islamic banks due to increased risk and moral hazards prevailing within society (Beck et al., 2013).

Firms seemed to have greater reliance over debt-based Islamic financing contracts, the reason being that it limits the agency problems (Alqahtani, 2016; Ebrahim & Sheikh, 2016; Shaban et al., 2014). The capital structure of the corporate sector is one of the determining elements of evolving agency conflicts. Naturally, non-PLS contracts are designed in such a way that it protects the firms’ interest in fixing the cost of financing at the time of contract. This feature is interest-based financing, which is unavailable and leads the managers to choose Islamic financing products. Our finding supports the above stated argument, which showed the significant effect on Industrial output (also, consistent with Guizani and Ajmi (2021)). Although our finding supports the previous literature, there is another important aspect that requires serious attention from the policy makers, i.e., social welfare and the creation of wealth. As per the literature, one of the seminal studies argued that non-PLS financing is sometimes considered as a back door for interest-based debt (Usmani, 2007). Due to personal influence, the valid process flow of conducting Shariah compliant contracts is often compromised, which gives rise to an identical interest-based loan (Atal et al., 2022). In addition, the essence of Islamic finance is compromised if banking companies focus on debt-based financing (non-risk sharing), as the socio-economic objective is ignored due to the lower emphasis on PLS financing (Usmani, 2007).

#### Control variables

Surprisingly, the impact of inflation on industrial production turned out to be significantly positive. This result evidenced the presence of a threshold effect (Khan & Senhadji, 2001). It implies that the rate of inflation remains within the threshold limit. Furthermore, the rate of inflation stayed within the low and medium level because of the quarterly data used in the study. Creeping inflation is healthy for an economy, even though the level of inflation is different from the economic state of a country. In this case, the threshold limit for developing countries has been reported as less than 9–11% (Khan & Senhadji, 2001). Model (2), (3) for PLS, and (6), (7) for non-PLS successfully predicted that prevailing inflation within the selected countries is healthy for promoting real economic activities. In our final model, we found that exports did not affect industrial production significantly. The coefficients for exports are -0.022 and 0.0353 for PLS and non-PLS models, respectively. The plausible explanation for this is gathered from a study (Saltarelli et al., 2020), which states on that aggregate level exports represent a good proxy for industrial production. However, the relationship between exports and production may decline due to the differences of exports of services and tangible products. The cross-country difference and specific class of exports, i.e., from service sector, may affect the theoretical basis in the export-production nexus. Furthermore, theoretically, imports are expected to have a negative and exports a positive relationship with industrial production. Imports are found to have the expected negative relationship but insignificant results with a coefficient of -0.00144 and -0.00155 for PLS and non-PLS, respectively. The results imply that an increase in imports discourages locally manufactured products, ultimately reducing the demand for local products, and as a result firms production fell (Suri & Chapman, 1998). Capital formation appeared to have a positive and significant impact on industrial production with a coefficient of 0.278 and 0.185 for models (3) and (7), respectively, i.e., significant at 1%. It implies that the flow of capital and investing activities enables producers and industries to buy the raw materials and industrial goods in advance, which will have a significant impact on real economic activity. Furthermore, this result also corroborates the findings of (Sankaran et al., 2020).

#### Moderating effect of asset quality

We introduced an interaction term of financing and NPA to capture the moderating effect of asset quality between financing and output nexus. NPA measured by non-performing assets/total financing by Islamic banks. A lower value of NPA would represent a strict compliance credit and recovery policy by Islamic banks. Furthermore, Islamic financial contracts naturally provide hedging against default due to stringent Shariah compliance. The negative coefficients of NPA − 13.94 and − 32.40 in models (3) & (7), respectively, are significant at 1%. This shows lower NPA, which refers to a lower ratio of non-performing loans in Islamic banks. This finding confirms the previous findings that Islamic banks are better at mitigating risk and better in asset quality (Alsamara et al., 2019; Hassan & Hussein, 2003; Mensi et al., 2020). However, our results found a positive and significant moderating effect flowing from assets quality to industrial production. The effect of Islamic financing magnified the effect of Islamic financing on economic activity. The positive and significant coefficients in both models (0.772 and 2.228, significant at 1%) confirm our second hypothesis of a moderating effect of NPA. The results infer that timely recovery of receivables is not only linked to the growth of assets for Islamic banks (Pratama, 2019), but for firms as well. Companies paying their debt obligation in time enhance their credit worthiness, leading to less financial constraints in future. Furthermore, a sound credit policy is cyclical in loan growth, so a bank with better credit default history would attract more corporate clients (Bordalo et al., 2018). Similarly, banks maintain internal and external credit ratings for their customers. Firms with lower credit rating tend to have more financial constraints than the ones who have a better rating (Bottazzi et al., 2014; Laghari & Chengang, 2019). Hence, better asset quality enhances the aggregate economic output by allowing firms to run their operation smoothly, with assurance of easing credit to the private sector.

### Robustness check and other estimations

Robustness of results is checked in 3 ways. Firstly, we checked the robustness by introducing savings as another control variable. The effect of PLS and non-PLS financing remained the same in robustness checks as shown in Table 5 columns 4 and 8, respectively. Compared to the main model, robustness checks produced stronger coefficients for both financing modes. We added the same robustness models in Table 6, along with other estimations. The relationship between Islamic financing and economic output may also be affected by endogeneity. In addition, panel models are often diagnosed with problems of endogeneity mainly for two reasons: omitted variable bias and reverse causality (Leszczensky & Wolbring, 2022). These are possible problems that may cause the endogeneity (Barros et al., 2020; Greene, 2003).

**Table 6:** Robustness and alternate analysis

	(I)	(II)	(III)	(IV)	(V)	(VI)	(VII)	(VIII)
Variables	IP Robust	IP Reverse	PLS First-step	2SLS Second-step	IP Robust	IP Reverse	non-PLS First-step	2SLS Second-step
			PLS				non-PLS	
PLS/nPLS	0.00828*	–	–	–	0.154***	–	–	–
	(1.974)				(7.914)			
PLS/nPLS(–1)	–	0.0297**	–	–	–	0.0161*	–	–
		(2.472)				(1.918)		
Fitted (PLS/nPLS)	–	–	–	0.330	–	–	–	6.843***
				(1.901)				(7.559)
Dep	− 0.0350	–	− 0.104**	–	0.0302	–	0.400*	–
	(− 1.608)		(− 2.376)		(0.113)		(1.992)	
Ex	0.00102	0.814***	− 0.254	− 0.0835***	0.0514	0.0171	− 0.223**	1.551***
	(0.0330)	(14.30)	(− 0.760)	(− 2.641)	(1.414)	(0.581)	(− 2.560)	(7.564)
INF	− 0.307	8.376***	12.37*	3.854***	− 1.238***	1.566***	4.070**	− 28.61***
	(− 0.511)	(4.662)	(1.900)	(8.109)	(− 3.487)	(3.381)	(2.399)	(− 7.115)
CF	0.522***	0.912***	− 1.055	0.144**	− 0.479***	− 0.783***	0.988	− 0.119*
	(3.377)	(4.797)	(0.569)	(2.014)	(− 5.224)	(− 2.214)	(4.574)	(− 1.988)
GFCF	0.401***	0.619***	0.286	0.496***	0.300***	0.214***	0.476***	− 2.931***
	(6.356)	(12.81)	(0.423)	(7.444)	(5.370)	(3.664)	(2.702)	(− 6.976)
Im	0.00969	− 0.334***	− 0.494	− 0.157***	0.00342	0.00855	0.0624	− 0.415***
	(0.327)	(− 4.233)	(− 1.533)	(− 4.511)	(0.150)	(0.303)	(0.742)	(− 6.742)
NPL	− 11.31***	20.73***	−	− 11.97***	− 24.20***	− 42.28***	–	− 36.96***
	(− 3.060)	(4.591)		(− 3.251)	(− 6.061)	(− 11.26)		(− 10.49)
PLS/nPLS*NPA	0.635**	− 1.561***	–	0.703**	1.682***	2.897***	–	2.556***
	(2.040)	(− 4.878)		(2.276)	(5.991)	(10.17)		(9.626)
Constant	21.33***	13.23***	14.13*	26.10***	19.75***	21.41***	9.879***	− 47.27***
	(31.00)	(40.26)	(1.952)	(33.24)	(27.61)	(35.96)	(5.236)	(− 5.178)
Year effect	Yes	Yes	Yes	Yes	Yes	Yes	Yes	Yes
Country effect	Yes	Yes	Yes	Yes	Yes	Yes	Yes	Yes
Observations	440	395	395	395	440	395	395	395
R-squared	0.790	0.528	0.483	0.690	0.794	0.449	0.579	0.534
F	1581	205.4	146.4	1666	6741	33.63	888.3	47.32
Cragg–Donald	–	–	11.57	–	–	–	15.06	–
Sargan test (*p* value)	–	–	–	0.124	–	–	–	0.113

***p < *0.01*, **p < *0.05*, *p < *0.1*

We addressed these issues by considering two separate models. The reverse causality is addressed by introducing the lagged independent variables (PLS and non-PLS) on IP. Omitted variable bias is countered by applying a two-stage least square regression (2SLS). It is quite challenging to capture the impact of all possible determinants of IP in a real-world scenario, which may lead to omitted variable bias. However, considering the theory of financial intermediation (Gurley & Shaw, 1966; Keynes, 1936), the financing side of banking is determined by deposits of other people. In other words, deposits are one of the main determinants of the financing side of banks; hence, financings are endogenously determined by deposits (Masood & Ashraf, 2012). Given this scenario, it is possible that the finance-economic output relationship is determined simultaneously with reverse causality, and independent variables are endogenously determined. Addressing these issues, we further checked the robustness of the selected model with two further models.

#### Reverse causality

To check if our model is not affected by reverse causation, we regressed our model with a lagged value of PLS and non-PLS against the current year IP. Keeping all other variables, year and country fixed, the results are provided in Table 6 column (II) and (VI) for PLS and non-PLS, respectively. Both the models showed a positive effect (0.0297 and 0.0161) and significant (5% and 1%) flowing from the financing side to industrial production. This indicates a causality flow from the financing side of Islamic banks to firm’s production capacity but not vice versa.

#### Alternate approach (2SLS approach)

We applied an instrumental variable approach by using two-stage least square regression (2SLS). 2SLS addresses two issues: first, it provides an alternate estimate of our selected model; second, it deals with endogeneity issues. In the model for checking robustness, we used savings as an additional control variable. We found the deposits/saving pattern does not appear to affect Industrial production. On the other hand, as per theory (Gurley & Shaw, 1966; Keynes, 1936), lending behaviors of banks are mainly determined by deposit structures. In addition to the above argument, we expect deposits to be endogenous to PLS and non-PLS financing in selected models.

The selection of an instrumental variable (I.V.) is conditional to two requirements, i.e., it does not influence dependent variable (IP) except through the independent variable (PLS/non-PLS) (Iwashyna & Kennedy, 2013). The relevance condition (correlation with PLS/non-PLS) and condition of exclusion restriction (I.V. affects IP only through independent variable) must be met. We regressed PLS and non-PLS financing against deposits with the inclusion of control variables (Column III and VII), which represent the first-stage regression. The coefficient of deposits is statistically significant in first-stage regression for PLS (0.104) and non-PLS (-0.400). This shows that deposits predict the Islamic financing side significantly. However, the validity of the selected instrument was further confirmed by the F statistics of first-stage regression and the Cragg–Donald Wald (Cragg & Donald, 1993) F value, which is greater than 10% (Stock & Yogo, 2005). Fitted independent variables from first-stage regression are generated and employed in the second-stage regression (column IV (PLS-fitted) and VIII (non-PLS-fitted)). To ensure the violation of over-identification, we run Sargan (1958) test, which gave insignificant results (p value is more than 5%). The results are consistent with the findings of the base regression model. However, fitted PLS has a positive (0.330) but insignificant coefficient (t-stat 1.901). Based on the estimate techniques, the results are robust with different econometric models and not driven by the issue of endogeneity.

We applied several diagnostic tests to make sure the model applied a good fit and does not provide spurious results. Diagnostic tests for multicollinearity, autocorrelation, and heteroskedasticity were performed and gave satisfactory results. As shown in Tables 5 & 6, the LM test (Engle, 1984), Breusch–pagan, and Chi-square produced favorable results.

## Conclusion

The current study aimed to shed light on the importance of Islamic financing modes and their contributory role in channelizing funds to enhance real economic activities within the economy. Furthermore, the presence of financial instability within the Islamic banking system is also accounted for in this study. For the analysis, 11 countries were selected based on their highest contribution to the global and domestic Islamic financial assets. Quarterly data were collected from the Islamic Financial Services Board (IFSB), International Monetary Fund (IMF), and the World Bank database. Based on the nature of the dataset, three possible Panel regression models were employed, and the best fit model was selected based on diagnostic tests.

The findings highlighted three key insights into Islamic financial development and economic growth. We concluded that the majority of the countries both the financing tools are important for smooth functioning of economic activities. The result further implied that Islamic banks are reluctant to offer funds in profit and loss sharing modes to finance the industries due to increased risk involved. On the other hand, sales-based modes appeared to play a key catalytic role in accelerating economic activities. The major contribution comes from Murabaha (sale on disclosed profit) and Ijarah (rental contract). Additionally, the investors and Islamic banks are reluctant to invest in risk-sharing modes as it contains a higher level of risk compared to non-risk sharing modes such as Murabaha, Ijarah, Istisna, and Salam. Furthermore, non-PLS modes act as a hedging tool in protecting companies’ agency problems as it removes the asymmetries in financing contracts. Our findings provide a generalized result by choosing a wide range of selected countries. Together with our findings, we provided a novel finding of credit risk and asset quality. Better asset quality complements the relationship of finance-growth.

The results provide implications for the regulatory bodies, and the management of Islamic banks gives importance to the profit and loss sharing modes as a financing tool and to encourage investors to utilize funds in these modes. Importantly, the biggest criticism of Islamic banking is its dependence on non-risk sharing modes of financing. This flaw can be overcome if the prime focus is given to PLS financing on a corporate level. Moreover, it will give rise to entrepreneurial innovation (Chandler, 2022) within the economy. Together with our findings, this paper also gives insights into asset quality serving as a factor of financial instability, which halts the progress of economic activities. Macroeconomic factors including imports and exports have a confounding and complementary relationship with industrial output, respectively. However, importantly, inflation has been found to be healthy for economic prosperity and the presence of a threshold effect evidenced from our results.

Although this study provides the first evidence of decomposed Islamic financing on economic output on a large set of countries, still literature can be enriched in this domain in following ways. Out of 15 countries with major contributions in Islamic financial assets, only 11 countries were considered in this study due to a limitation on data available. If the data are available of all the countries with an Islamic banking system, the results can provide better insights on this subject. Furthermore, only Sudan and Iran have fully implemented an Islamic financial system, but due to a lack of availability of data we could only include Iran in this study. However, countries with 100 percent Islamic banking systems must be studied to give insights into the other countries, to implement Islamic financial system successfully. Further research can be carried out by increasing the sample size and by including the data from Islamic windows, and sukuk market as well. In addition, the corporate side and their appetite for PLS and non-PLS financing need to be addressed in future. This could help bridge the gap between corporate sector needs and the finance-growth nexus. As a result, the countries with a majority Muslim population and a relatively lower share of Islamic finance can trace out the growth opportunities to expand its market share. The religiosity factor of selection can play a vital role in filling this gap. If the selection of debt products is chosen based religious faith, the researcher may uncover the reasons why Muslim majority countries are still lagging behind in terms of Islamic financial solutions.

## Data Availability

This study is based on a secondary dataset. All the data are available on Financial Service Board (IFSB) (https://www.ifsb.org/psifi_06.php), International financial statistics (IFS) (https://data.imf.org/?sk=4C514D48-B6BA-49ED-8AB9-52B0C1A0179B), and World bank dataset (WDI) (https://databank.worldbank.org/source/world-development-indicators).
